# Raman Spectroscopy Study on Chemical Transformations of Propane at High Temperatures and High Pressures

**DOI:** 10.1038/s41598-020-58520-7

**Published:** 2020-01-30

**Authors:** Daniil A. Kudryavtsev, Timofey М. Fedotenko, Egor G. Koemets, Saiana E. Khandarkhaeva, Vladimir G. Kutcherov, Leonid S. Dubrovinsky

**Affiliations:** 1 KTH Royal Institute of Technology, Stockholm, Sweden; 2 University of Bayreuth, Bayreuth, Germany; 3 Gubkin Russian State University of Oil and Gas (National Research University), Moscow, Russia

**Keywords:** Applied physics, Chemical physics, Condensed-matter physics, Structure of solids and liquids, Organic chemistry, Physical chemistry

## Abstract

This study is devoted to the detailed *in situ* Raman spectroscopy investigation of propane C_3_H_8_ in laser-heated diamond anvil cells in the range of pressures from 3 to 22 GPa and temperatures from 900 to 3000 K. We show that propane, while being exposed to particular thermobaric conditions, could react, leading to the formation of hydrocarbons, both saturated and unsaturated as well as soot. Our results suggest that propane could be a precursor of heavy hydrocarbons and will produce more than just sooty material when subjected to extreme conditions. These results could clarify the issue of the presence of heavy hydrocarbons in the Earth’s upper mantle.

## Introduction

Thermal and catalytic transformations of various hydrocarbon compounds at normal pressure have attracted significant attention in the field of petrochemistry. However, high-pressure chemistry of hydrocarbons as a science started to develop only recently, —primarily due to the unavailability of the specific equipment for the experiments. To date, only methane, the first member of the alkane homologous series, has been investigated when subjected to a wide range of pressures and temperatures^1–4^, because of its widespread occurrence in geological systems^5–7^ and well-known role in the atmosphere of the Solar System’s outer planets^8,9^. The behavior of other hydrocarbons, both unsaturated^10,11^ and saturated^12,13^, have been less widely investigated with the use of various high-pressure techniques.

The focus on the significance of methane’s high-pressure high-temperature behavior implies that the fate of higher hydrocarbons has been ignored. Though the high-pressure, high-temperature (HPHT) behavior of ethane has been investigated several times^12,14^, propane has only been studied at ambient temperatures^15,16^. Propane is the third most abundant hydrocarbon on Earth after methane and ethane. It has been detected in the atmosphere of outer planets^17^ and their satellites^18^, and is a typical product of HPHT hydrocarbon synthesis performed both for chemical and geological purposes^19,20^.

The relevance of the investigation of carbon-bearing compounds can be understood from the perspective of the growing evidence of the role of hydrocarbon compounds deep in the Earth’s interior, which could contribute to the global carbon cycle^21,22^. Unfortunately, even for methane, investigations into its behavior under conditions of high pressure have yielded inconclusive and mutually conflicting results.

Propane’s importance as a petrochemical feedstock led to detailed studies of its thermal transformations in the range of 500–900 °C in processes such as pyrolysis and thermal cracking^23–25^. By changing the basic conditions of the process, the content of hydrocarbon compounds complex systems could be varied from higher normal and isoalkanes, dienes, arenes, and alkenes to C_1_-C_3_ fractions. These thermal processes were only investigated in the diapason under relatively mild pressures because of the process goal—low pressures are favorable for the synthesis of low-molecular compounds, while higher pressures could cause secondary reactions, particularly, polymerization and condensation, to occur^25^.

Considering the previous information, this study deals with the HPHT study of propane under a pressure range of 2–22 GPa and a temperature range of ~900–3000 K.

## Methods

Propane (Linde Gas Polska), with a purity of 99.99%, was used in the experimental procedure without any additional purification. In our experiment, propane was subjected to cooling by liquid nitrogen and subsequent cryogenic loading in symmetric BX-90-type diamond anvil cells (DACs) equipped with synthetic, CVD-type IIa diamonds with a culet size of 250 μm. The rhenium gaskets were indented to a thickness of 25 μm. Pressure chambers with a step (Fig. S1 in supplementary) were prepared in the gaskets by combination of laser ablation and drilling. Thin (~1–2 μm) gold foil act as heat absorber in experiments with laser heating.

The Raman spectra were excited using a He-Ne laser (632.8 nm excitation). Then the the acquisition of the spectra was made via the use of a LabRam spectrometer with a 2 cm^−1^ spectral resolution. If possible, the pressure was determined by a calibration of propane high-pressure behavior^16^, or else the pressure was determined by the first-order peak of the diamond. The uncertainties in the Raman peak positions were ±1 cm^−1^. Raman spectra were collected at several points of the heating areas to ensure that the transformations being investigated, actually occurred. The Raman spectra of propane and the products of the reaction were measured before and after heating under the required thermobaric conditions.

In some cases, the green Ar^+^-laser (514.5 nm) the LabRam spectrometer (2 cm^−1^ spectral resolution) was equipped with was also employed for the *in situ* analysis.

The laser heating of the samples was performed using a home-laboratory laser heating setup at the Bayreuth Geoinstitut^26^. This system could be described as transferable double-sided laser heating setup for diamond-anvil cells with the possibility of *in situ* temperature determination and precise heating of the samples inside a cell. Using high magnification and low working distance infinity corrected laser focusing objectives provided the opportunity of the laser beam size decrease less than 5 μm as well as achievement of the 320 times optical magnification.

Heating of the sample is carried out by two YAG lasers (1064 nm central wavelength). For temperature measurements the thermal emission spectra of the heated area is guided into an IsoPlane SCT 320 spectrometer with a 1024 × 256 PI-MAX 4 camera. The temperature was determined by fitting the black body radiation spectra of the heated area in a given wavelength range (570–830 nm) to the Plank radiation function. Liquid and solid propane are optically transparent and do not absorb well at the central wavelength of the YAG laser. This means that it is important to find a way to heat the sample and eliminate the catalytic influence of the absorber that could appear because of the usage of noble metals such as Ir. For these reasons, gold foil was employed as the absorber of the laser radiation to dissipate heat to the sample. The Raman spectra were measured at the hot points, near the hot points (marked as “near” on the several spectra), as well in the cold sample areas to facilitate a deeper understanding of propane’s behavior.

## Results and Discussion

### 3GPa

**Figure 1:**
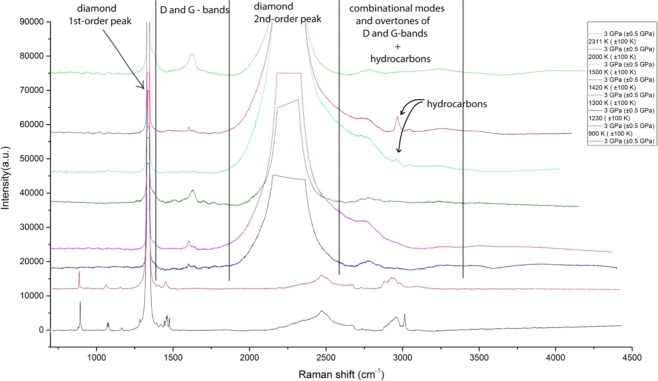
Chemical transformations of propane at 3 GPa and T = 900–2300 K (±100 K). The reference peaks for graphite (soot) modes were taken from^27,28^, for C-H valence of hydrocarbon compounds^2–4,12^. The propane remained stable at 900 K. The spectra of untouched propane are in good correspondence with the previous experiments we carried out^37^.

The main chemical transformation of propane at 3 GPa (Fig. 1) observed at the temperatures displayed by Fig. 1 is a reaction with the prevalent formation of a sooty material. This material has very similar Raman spectra to the typical black solid compound obtained during the thermal and catalytic petrochemical processes or as a by-product of combustion according to the reaction:

$${{\rm{C}}}_{3}{{\rm{H}}}_{8}=3{\rm{C}}+4{{\rm{H}}}_{2}$$

During such processes, there is also the possibility of obtaining a graphite, which could be either disordered or highly ordered. However, the character of the presented spectra of the propane reaction products indicates the presence of disordered phases of graphite or soot^27^. The highly ordered Raman spectra of graphite exhibits only one band (first-order G-bands) at 1580 cm^−1^ at ambient temperature. On the contrary, the disordered structure of graphite has the presence of additional first-order bands (D-bands) at 1360 and 1620 cm^−1^ depending on the ambient conditions^28^. The bands in the region of 2800–3500 cm^−1^ could also correspond to the combinational models of D and G-bands^26,27^. The signals of the graphite and soot are hard to distinguish, however, there is evidence that the soot itself has broader peaks^27,28^. The spectra of propane under pressure of 3 GPa and at an ambient temperature of 900 K (±100 K) show no major changes in the display of any of the bands that are typical for propane^16^. The stability of propane at a temperature of ~900 K is in good correspondence with the earlier experiments of Kolesnikov *et al*.^12^ on methane and ethane, showing the same behavior of propane. The absence of the hydrogen peaks in the region of 500–800 cm^−1^ (the region was not shown on the graph) can be explained as being due to the high rate of hydrogen diffusion through the rhenium gasket or through the reaction products. The appearance of the intense peak at ~3000 cm^−1^ at temperatures of 1420 and 1500 K (±100 K) could be attributed to C-H vibrations of various aliphatic hydrocarbons due to the well-known radical reaction mechanism resulting in the formation of methyl and ethyl radicals. These radicals could subsequently react via various pathways leading to the formation of hydrocarbon compounds^29^:

$$\begin{array}{c}\begin{array}{c}{{\rm{CH}}}_{3}-\,{{\rm{CH}}}_{3}-{{\rm{CH}}}_{3}\\ {\rm{propane}}\end{array}\to \mathop{{{\rm{CH}}}_{3}{\dot{{\rm{C}}}{\rm{H}}}_{2}}\limits_{{\rm{ethyl}}\,{\rm{radical}}}+\mathop{{\dot{{\rm{C}}}{\rm{H}}}_{3}}\limits_{{\rm{methyl}}\,{\rm{radical}}}\\ \begin{array}{c}{{\rm{CH}}}_{3}-{{\rm{CH}}}_{2}-{\dot{{\rm{C}}}{\rm{H}}}_{3}\\ {\rm{propane}}\end{array}\to \mathop{{{\rm{CH}}}_{4}}\limits_{{\rm{methane}}}+\mathop{{{\rm{CH}}}_{3}-{{\rm{CH}}}_{2}-{\dot{{\rm{C}}}{\rm{H}}}_{2}}\limits_{{\rm{propyl}}\,{\rm{radical}}}\\ {{\rm{CH}}}_{3}-{{\rm{CH}}}_{2}-{\dot{{\rm{C}}}{\rm{H}}}_{2}+{\dot{{\rm{C}}}{\rm{H}}}_{3}\to \mathop{{{\rm{CH}}}_{3}-{{\rm{CH}}}_{2}-{{\rm{CH}}}_{2}-{{\rm{CH}}}_{3}}\limits_{{\rm{n}}-{\rm{butane}}}\\ {{\rm{CH}}}_{3}{\dot{{\rm{C}}}{\rm{H}}}_{2}+{{\rm{CH}}}_{3}-{{\rm{CH}}}_{2}-{{\rm{CH}}}_{3}\to \mathop{{{\rm{CH}}}_{3}-{{\rm{CH}}}_{3}}\limits_{{\rm{ethane}}}+{{\rm{CH}}}_{3}-{{\rm{CH}}}_{2}-{\dot{{\rm{C}}}{\rm{H}}}_{2}\\ {{\rm{CH}}}_{3}{\dot{{\rm{C}}}{\rm{H}}}_{2}+{{\rm{CH}}}_{3}-{{\rm{CH}}}_{3}-{\dot{{\rm{C}}}{\rm{H}}}_{2}\to \mathop{{{\rm{CH}}}_{3}-{{\rm{CH}}}_{2}-{{\rm{CH}}}_{2}-{{\rm{CH}}}_{2}-{{\rm{CH}}}_{3}}\limits_{{\rm{n}}-{\rm{pentane}}}\end{array}$$

Above mentioned reaction pathway may also explain why we do not observe pure hydrogen in the system. Hydrogen could be consumed in the reactions with other hydrocarbons:

$${{\rm{C}}}_{3}{{\rm{H}}}_{8}+2{{\rm{H}}}_{2}=3{{\rm{CH}}}_{4}$$

### 6GPa

**Figure 2:**
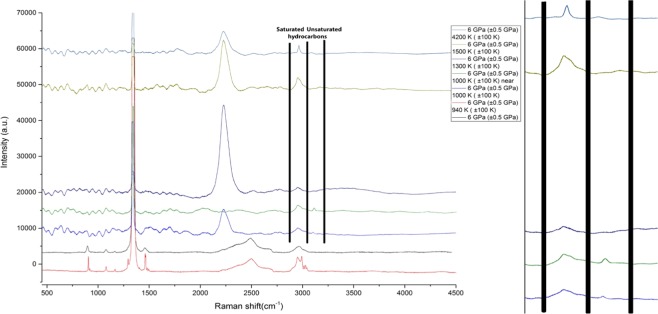
Chemical transformations of propane at 6 GPa and T = 940–1600 K (±100 K). The reference peaks for C-H vibrations of saturated hydrocarbon compounds were taken from^2–4,12^, and for unsaturated hydrocarbons from^30,32^. The strong fluorescence in the region of the hydrocarbon footprint is explained by the presence of complex hydrocarbon systems having a mixed structure. The formation of ultradispersive diamonds could also affect the spectra. The propane remained stable at 940 K. The spectra of pristine propane are in good correspondence with the previous experiments that we carried out^37^. On the right side of the figure there is the magnified region of the C-H valence vibrations of the saturated and unsaturated hydrocarbons.

Heating at 940 K does not affect Raman spectra of propane (Fig. 2).

The spectra collected after heating at higher temperatures characterized by presence of bands at ~3100–3200 cm^−1^ (may be attributed to formation of unsaturated compounds^2–4,12^), and peak at ~3000 cm^−1^ (probably due to saturated hydrocarbon(s)). It is impossible to attribute these peaks to an individual compound or group of compounds due to high fluorescence in C-C stretching region or due to formation of ultradisperse diamonds^30^. We hypothesize that due to complicated thermal mechanisms of propane transformations the products of polymerization or aromatization were obtained, for instance, via allyl-radical reaction:



It is important to notice, that in the works of Kolesnikov *et al*.^31,32^ the formation of unsaturated hydrocarbons wasn’t reported. Propane is heavier than methane by mass, and thus propane can easier decompose and produce larger number of hydrocarbon compounds.

### 8GPa

**Figure 3:**
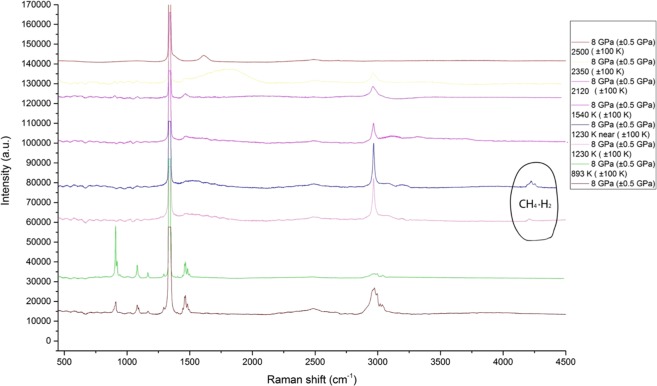
Chemical transformations of propane at 8 GPa and T = 893–2500 K (±100 K). The reference peaks for C-H valence of saturated hydrocarbon compounds were taken from^2–4,12^, for unsaturated hydrocarbons, they were obtained from^30,32^, and for graphite (soot) modes, they were taken from^27,28^. The strong fluorescence in the region of the hydrocarbon footprint is explained by the presence of complex hydrocarbon systems of mixed structure. The possible formation of ultradispersive diamonds could also affect the spectra. The propane remained stable at 893 K. The spectra of untouched propane are in good correspondence with the previous experiments carried out by us^37^. The complex methane-hydrogen compounds reference peaks were obtained from^33^.

With the increase of the pressure to 8 GPa only methane could be identified among all of the hydrocarbons along with the formation of complex compounds with hydrogen at 1230 K (Fig. 3). These are formed due to the escape of hydrogen from the reacting system and the consequent formation of Van Der Waals bonds^33^.

Unfortunately, the region of the hydrocarbon footprint lacks the characteristic peaks overlapped by the fluorescence. However, the C-H of aliphatic and unsaturated fragments in the region of valence vibrations suggests the presence of various hydrocarbon compounds. With the increase of the temperature up to 2000 K and higher, the formation of graphite-like systems could be seen with the total disappearance of C-H and C-C vibrations at 2350 K.

Another possible mechanism of hydrocarbon generation as well as hydrogen could be the interaction of various forms of carbon and hydrocarbons. There is evidence that C-H fluids could be in contact with carbon^34^ in the Earth’s mantle, which could lead to certain chemical reactions of hydrogenation. For example, the hydrogen generation in our case could be explained not only by thermal decomposition of hydrocarbons, but by the catalytic effect of the carbon in the form of graphite or soot^35,36^.

### 11 and 14 GPa

The reference peaks for C-H valence of saturated hydrocarbon compounds were obtained from^2–4,12^, for unsaturated hydrocarbons from^31,32^, for graphite (soot) modes, they were obtained from^27,28^. The reference peaks for C-C stretching and C-C bending of hydrocarbons were obtained for ethane from^12^, for propane from^12,37^, for n-butane from^12,37^, for n-pentane from^38^, and for n-hexane from^38^. The propane remained stable at temperatures of 840 K and lower. The spectra of untouched propane are in good correspondence with the previous experiments we carried out^37^.

**Figure 4:**
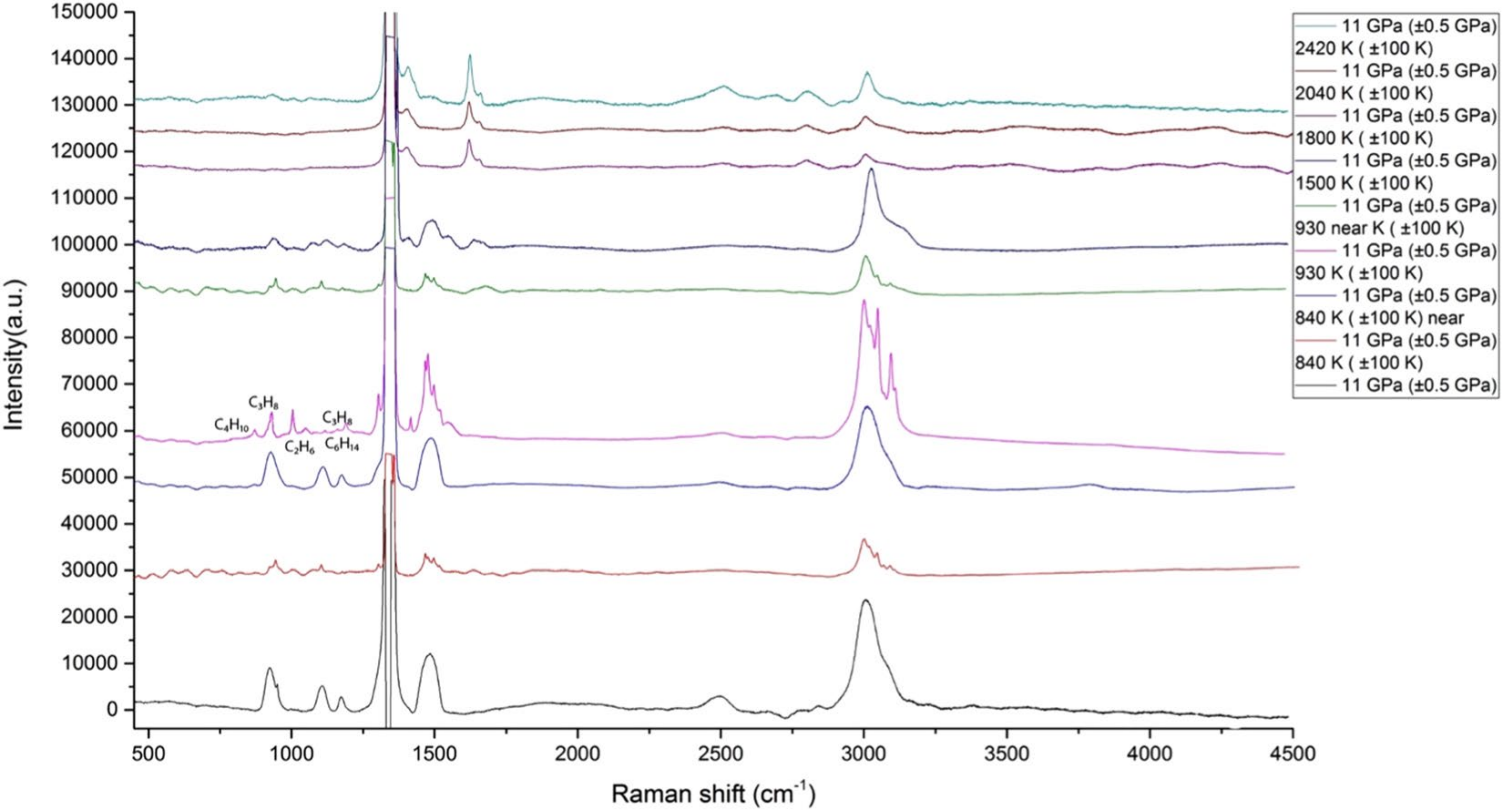
Chemical transformations of propane at 11 GPa and T = 840–2420 K (±100 K).

**Figure 5:**
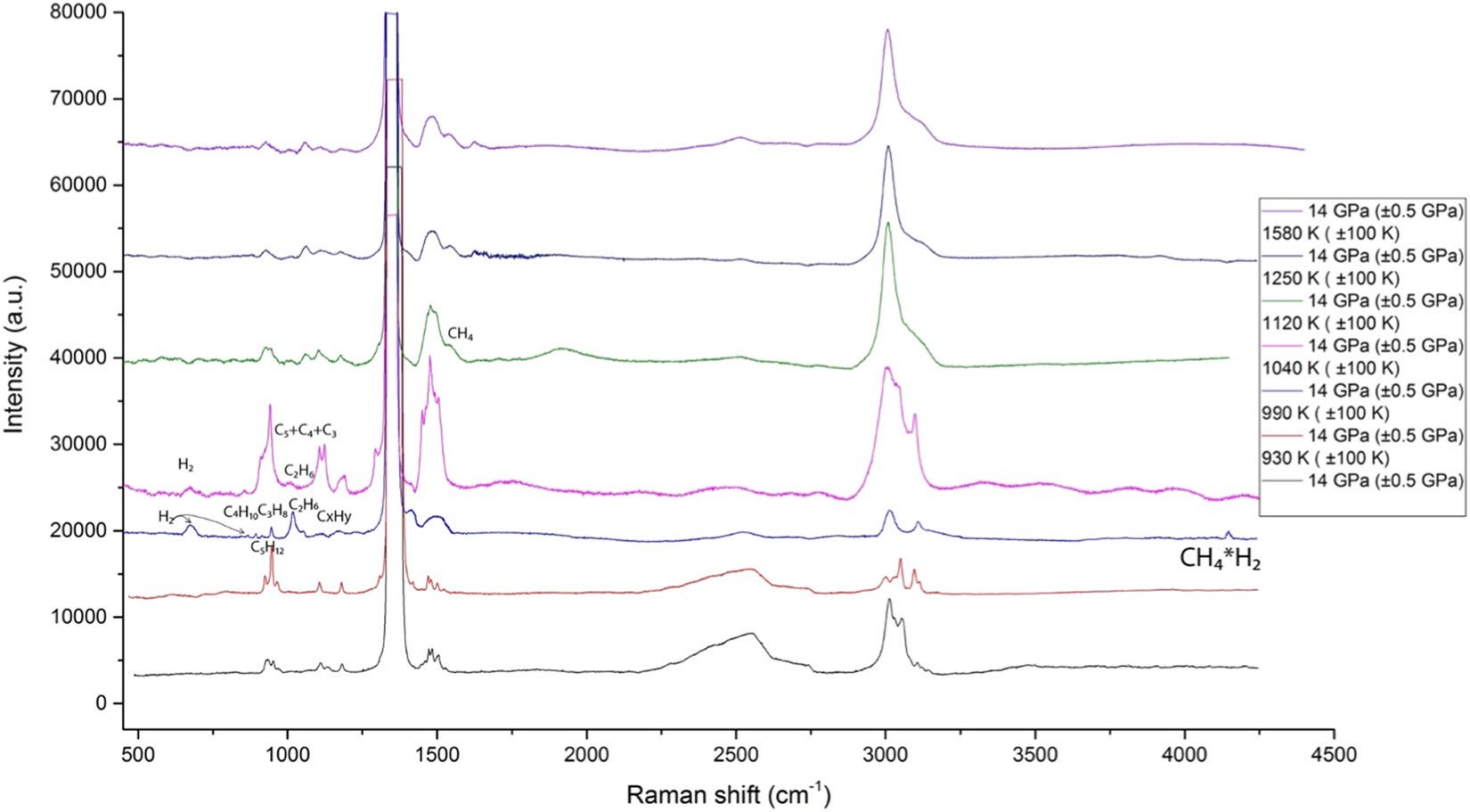
Chemical transformations of propane at 14 GPa and T = 930–1580 K (±100 K). The reference peaks for C-H valence of saturated hydrocarbon compounds were obtained from^2–4,12^, for unsaturated compounds the reference peaks were obtained from^31,32^, and for graphite (soot) modes, the reference peaks were obtained from^27,28^. The reference peaks for C-C stretching and C-C bending of hydrocarbons were obtained for ethane from^12^, the peaks for propane were obtained from^12,37^, the peaks for n-butane from^12,37^, the peaks for n-pentane from^39^, and the peaks for n-hexane were obtained from^38^. The propane remained stable at 930 K. The spectra of untouched propane are in good correspondence with the previous experiments we carried out^37^. The hydrogen vibrational modes were investigated in those experiments^33^.

The formation of C_1_-C_6_ hydrocarbons at 11 and 14 GPa (Figs. 4 and 5 respectively) starting from 900 K corresponds with the previous results for methane of Kolesnikov^12^ and is in good agreement with the results from simulation experiments^2^. The spectra of 11 and 14 GPa have a main, obvious difference—the presence of hydrogen. The absence of hydrogen at 11 GPa is because of hydrogen diffusion or secondary reactions of hydrocarbons or graphite. The C-C vibrations of n-butane^37^, n-hexane^39^, and n-pentane^38^ were detected in the spectra. In the case of the n-pentane and n-hexane, they were never detected in such types of experiments. This result proposes the complicated condensation mechanism that has a radical character, as in the case of industrial processes of pyrolysis. These series of reactions could be described with a classic radical-polymerization mechanism, because of a certain regularity in the decreasing intensity of the hydrocarbon peaks with the consequent increase in the molecular mass. By modifying the reaction of^12^, it is reasonable to assume that we obtain the following result:

$${{\rm{CH}}}_{4}\to {{\rm{C}}}_{2}{{\rm{H}}}_{6}+{{\rm{C}}}_{3}{{\rm{H}}}_{8}+{{\rm{C}}}_{4}{{\rm{H}}}_{10}+{{\rm{C}}}_{5}{{\rm{H}}}_{12}+{{\rm{C}}}_{6}{{\rm{H}}}_{14}\ldots +{{\rm{H}}}_{2}$$

The most important fact that was observed during the experiments at 14 GPa is the contemporaneous presence of hydrogen, graphite, and other hydrocarbons during the laser heating, which could be interpreted as the equilibrium state. However, with the pressure increase it is hard to distinguish the particular hydrocarbon signal. After 1500 K only graphite and C-H valence vibrations could be seen.

### 17 GPa and 22 GPa

**Figure 6:**
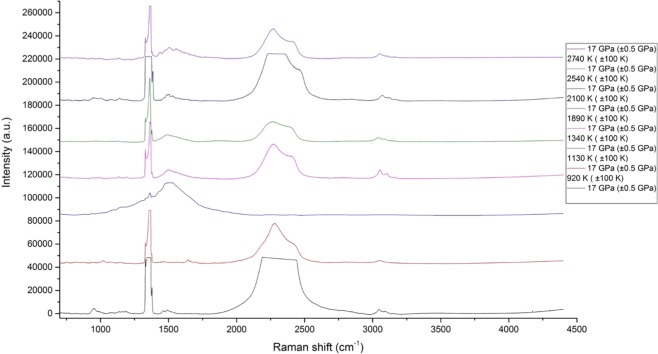
Chemical transformations of propane at 17 GPa and T = 920–3100 K (±100 K). The reference peaks for C-H valence of saturated hydrocarbon compounds were obtained from^2–4,12^, the corresponding reference peaks for graphite (soot) modes were obtained from^27,28^.

**Figure 7:**
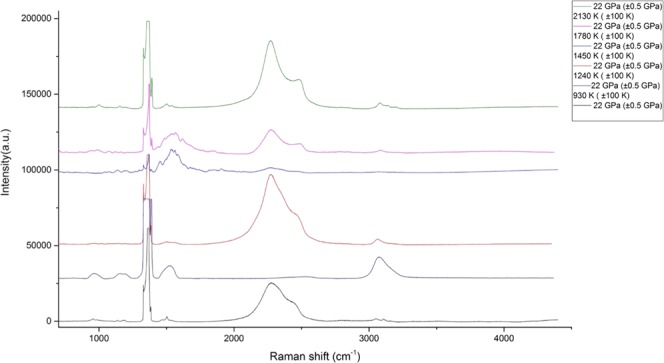
Chemical transformations of propane at 22 GPa and T = 930–2100 K (±100 K). The reference peaks for C-H valence of saturated hydrocarbon compounds were obtained from^2–4,12^, the reference peaks for graphite (soot) modes were taken from^27,28^.

At 17 and 22 GPa the Raman bands of hydrocarbons become less distinguished with the overlap of the C-C bending region by graphite frequencies with the presence of unidentified C-H vibrations of saturated hydrocarbons in the region of 3000 cm^−1^ (Figs. 6 and 7).

## Summary of The Results

**Figure 8:**
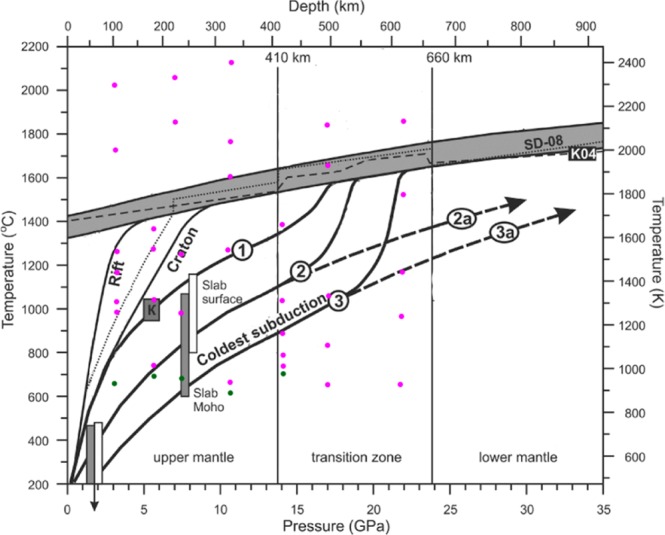
PT-range of hydrocarbon formation from propane in laser heating DAC experiments in comparison with mantle PT-profiles provided in the diagram taken from^42^. The pink dots represent the chemical transformations of propane, while the green – its stability.The gray field is the range of mantle adiabats with potential temperatures 1315–1415 °C. The dashed line represents the K04–1400 °C adiabat^43^. The dotted line depicts the average mantle thermal model^44^. 1- hottest subduction, 2- medium subduction, 3 – coldest subduction stagnant in the transition zone and penetrating into the lower mantle (2a and 3a)^45,46^.

The Table 1 and Fig. 8 summarize observations described above. Our experiments demonstrate that at pressure and temperature conditions relevant for wide range of depth within the Earth pure propane (without any catalyst) can transform in to different hydrocarbons, both saturated and unsaturated. Under these conditions propane is reacting via two simultaneous and competing pathways – (1) the growing of the hydrocarbon chain via condensation or polymerization mechanisms with the formation of higher hydrocarbons and (2) destruction via the cleavage of C-C and C-H bonds with the formation of lighter hydrocarbons and also graphite (or sooty material). Our observations suggest that propane, if subducted in to the mantle, undergoes complex transformations and may be source of more complex organic compounds.The issue of presence of heavy hydrocarbon compounds in the Earth’s mantle was thoroughly described and examined in these works^40,41^.

**Table 1:** Overview of the experiments carried out during the investigation.

Pressure	Temperature, K (±100 K)	Products
3 GPa	298	C_3_H_8_
	900	
	1230	C
	1300	
	1420	
	1500	Saturated hydrocarbons, C
	2000	
	2311	С
6 GPa	298	C_3_H_8_
	940	
	1000	unsaturated and saturated hydrocarbons
	1200	
	1300	
	1500	
	1600	
8 GPa	298	C_3_H_8_
	893	
	1230	C, unsaturated and saturated hydrocarbons, H_2_
	1230 near	
	1540	
	2120	C, saturated hydrocarbons
	2350	
	2500	C
11 GPa	298	C_3_H_8_
	840	
	840 near	
	930 near	
	930	C, CH_4_, C_4_H_10_, C_2_H_6_, C_3_H_8_, saturated hydrocarbons, C_6_H_14_
	1500	C, saturated hydrocarbons, CH_4_
	1800	
	2040	C, saturated hydrocarbons
	2420	
14 GPa	298	C_3_H_8_
	930	
	990	H_2_, CH_4_, C_4_H_10_, C_2_H_6_, C_3_H_8_, saturated hydrocarbons, C_5_H_12_
	1040	
	1120	CH_4_, saturated hydrocarbons, C
	1250	
	1580	
17 GPa	298	C_3_H_8_
	920	Hydrocarbons, С
	1130	
	1340	
	1890	
	2100	
	2540	
	2740	
22 GPa	298	C_3_H_8_
	930	Hydrocarbons
	1240	Hydrocarbons, С
	1450	
	1780	
	2130

## Conclusion

The observations of propane chemical transformations under a wide range of high pressures and temperatures that are also present in the Earth’s mantle and in subduction environments, provides an insight into the fate of the carbon-bearing fluids fate deep in the Earth’s interior. The thermodynamic stability of propane under the pressures of 3–14 GPa and temperatures less than 900 K have been shown. At temperatures greater than 900 K, over a full range of pressures, propane transformation led to the formation of complex hydrocarbon systems and soot. At pressures of 11 and 14 GPa it was possible to identify the product mixture which includes light hydrocarbons, methane, and ethane and heavy hydrocarbons such as n-butane, n-pentane, and n-hexane.

We also have shown that the formation of heavier alkanes from propane at temperatures in the range of ~1000–2000 K and under pressures from 6 to 22 GPa is possible without any catalysts, and corresponds to the reactions leading to the formations of similar compounds occurring at depths of more than 130 km beneath the Earth’s surface.

## Supplementary information

Supplementary information.
