# Supplementary material for: Raman Spectroscopy Study on Chemical Transformations of Propane at High Temperatures and High Pressures
Source: Sci Rep. 2020 Jan 30;10:1483. doi: 10.1038/s41598-020-58520-7 (PMC6992756; doi:10.1038/s41598-020-58520-7)
Supplement: Supplementary file 1 — Supplementary information. [file 41598_2020_58520_MOESM1_ESM.docx]

**Supplementary material**


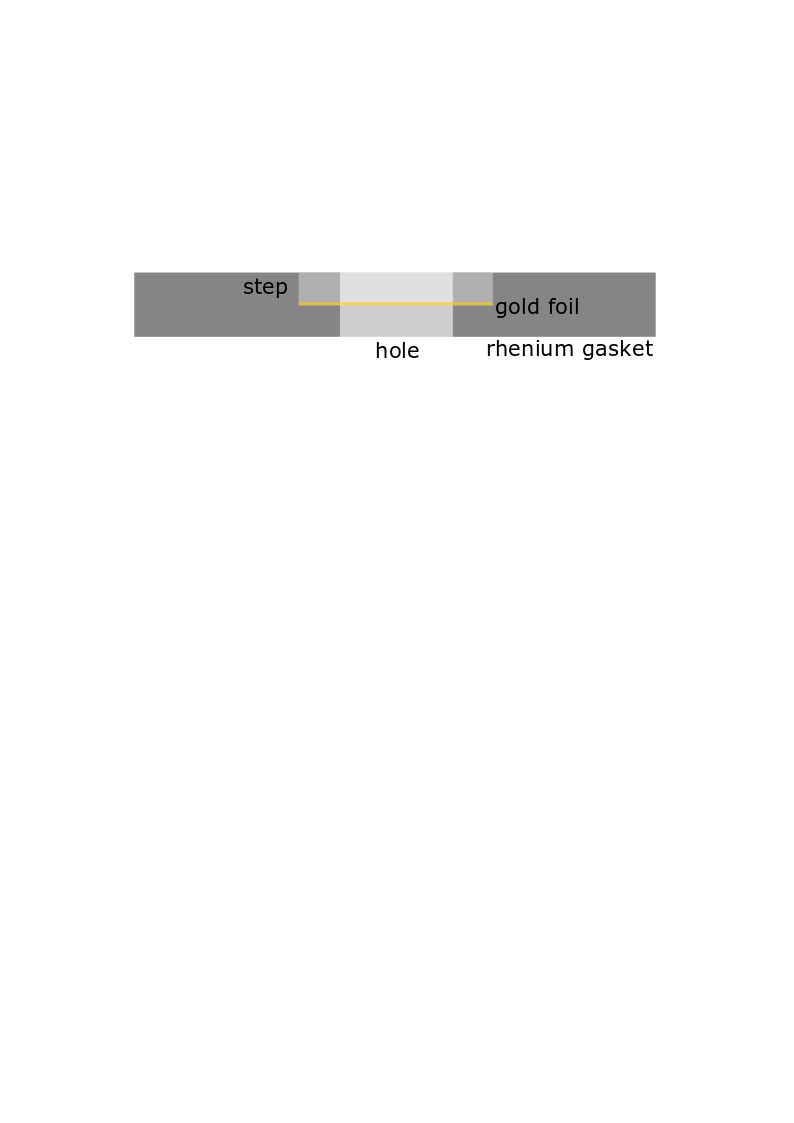


Figure S1. A gasket of 25 μm with the step of approximately a half of gasket thickness (12 μm) and diameter of 80 μm. The step was made by means of laser ablation technique. Then in the step the drilling of the 60 μm-diameter hole was made with the following placement of gold foil (~1-2 μm in thickness and 80 μm in diameter) inside the step.
